# Leucine-Rich Repeat Kinase 2 Is Associated With Activation of the Paraventricular Nucleus of the Hypothalamus and Stress-Related Gastrointestinal Dysmotility

**DOI:** 10.3389/fnins.2019.00905

**Published:** 2019-08-29

**Authors:** Tatsunori Maekawa, Hiromichi Tsushima, Fumitaka Kawakami, Rei Kawashima, Masaru Kodo, Motoki Imai, Takafumi Ichikawa

**Affiliations:** ^1^Department of Regulation Biochemistry, Graduate School of Medical Sciences, Kitasato University, Sagamihara, Japan; ^2^Department of Behavioral Medicine, Tohoku University School of Medicine, Sendai, Japan

**Keywords:** LRRK2, gut dysmotility, stress, paraventricular, signaling/signaling pathway

## Abstract

Leucine-rich repeat kinase 2 (LRRK2) is a molecule associated with familial and sporadic Parkinson’s disease. It regulates many central neuronal functions, such as cell proliferation, apoptosis, autophagy, and axonal extension. Recently, it has been revealed that LRRK2 is related to anxiety/depression-like behavior, implying an association between LRRK2 and stress. In the present study, we investigated for the first time the stress pathway and its relationship to gastrointestinal motility in LRRK2-knockout (KO) mice. The mice were subjected to acute restraint stress, and analyzed for activation of the paraventricular nucleus of the hypothalamus (PVN) using an immunohistochemical approach. Phosphorylation of extracellular signal-regulated kinase 1/2 (ERK1/2) was assessed by Western blotting. The KO mice showed a lower number of c-Fos-positive cells and disruption of the ERK signaling pathway in the PVN in the presence of restraint stress. Stress responses in terms of both upper and lower gastrointestinal motility were alleviated in the mice, accompanied by lower c-Fos immunoreactivity in enteric excitatory neurons. Our present findings suggest that LRRK2 is a newly recognized molecule regulating the stress pathway in the PVN, playing a role in stress-related gastrointestinal dysmotility.

## Introduction

Leucine-rich repeat kinase 2 (LRRK2) was originally defined by linkage analysis of familial Parkinson’s disease (PD), and its association with inflammatory bowel disease (IBD) and leprosy has been demonstrated recently (Funayama et al., 2002; Paisán-Ruíz et al., 2004; Barrett et al., 2008; Zhang et al., 2009). LRRK2 is a complex kinase comprising Leucine-rich repeat (LRR), Ras of complex (ROC), C-terminal of Roc (COR), kinase, and WD40 domains (Meylan and Tschopp, 2005). It has already been reported that LRRK2 is expressed in neurons, astroglia, and microglia in the central nervous system (CNS), and several types of immune cells (Higashi et al., 2007; Kubo et al., 2010; Liu et al., 2011; Moehle et al., 2012). Accumulated evidence suggests that LRRK2 plays a key role in endocytosis, axonal extension, autophagy, proliferation, and survival of neurons (Esteves et al., 2014; Maekawa et al., 2016a, b). While many studies have been focusing on the role of LRRK2 in the pathogenesis of motor dysfunction in PD, some groups have reported an association between LRRK2 and the subtle non-motor phenotypes of PD. It has been shown that LRRK2-knockout (KO) mice display anxiety/depression-like behavior (Hinkle et al., 2012). In addition, transgenic mice harboring the G2019S (glycine to serine substitution at amino acid 2019) mutation have been shown to display anxiety/depression-like behavior, despite the paradoxical phenotype of G2019S mutation gain of function and loss of LRRK2 in KO mice (Lim et al., 2018). These findings suggest an association between LRRK2 and stress.

Physiological or psychological stress activates several brain regions, including the amygdala, paraventricular nucleus (PVN), and bed nucleus of the stria terminals, followed by activation of the pituitary gland (Rutherford et al., 2011). This activation induces peripheral stress responses, such as increases in gluconeogenesis, vasoconstriction, and heart rate together with decreases in growth, immunity, and digestion via the sympathetic system and hormonal pathway (Panagiotakopoulos and Neigh, 2014). Restraint stress in rodents is a well-known model of typical stress that activates PVN followed by development of peripheral responses (Kwon et al., 2006; Zheng et al., 2009; Campos-Rodríguez et al., 2013).

Gastrointestinal dysmotility is a feature of PD as well as stress and psychiatric disease (North et al., 2007; Konturek et al., 2011; Pellegrini et al., 2015). While many clinical studies have been reported, few investigations have focused on the role of enteric neurons and brain-gut interaction in PD-related gastrointestinal dysmotility (Derkinderen et al., 2011). Furthermore, in contrast to accumulated data on the function of LRRK2 in the CNS, it is unclear whether LRRK2 is associated with regulation of the stress signaling pathway and gastrointestinal motility.

In the present study using LRRK2-KO mice, we have revealed for the first time that the extracellular signal-regulated kinase (ERK) signaling pathway is disrupted in the PVN and that stress suppresses gastrointestinal dysmotility, thus highlighting the role of LRRK2 in the regulation of stress signaling and gastrointestinal motility.

## Materials and Methods

### Animals

Male LRRK2-KO mice on C57BL/6J, developed by Hinkle et al. (2012), and littermate wild-type (WT) mice aged 8–14 weeks were used. The mice were housed in a light- and temperature-controlled room with water and food available *ad libitum*. For sacrifice, the mice were euthanatized by cervical dislocation. All procedures had been approved by the Animal Experimentation and Ethics Committee of Kitasato University.

### Restraint Stress

Single exposure to restraint stress for 60 min was performed by placing the animal in a 2.9 × 11.5-cm bottle, adjusting it with tape on the outside so that the animal was unable to move. 1 h after the end of stress exposure, the mice were sacrificed for each type of analysis.

### Immunostaining of Brain Sections

Mice were subjected to flush-perfusion with phosphate-buffered saline (PBS) followed by perfusion-fixation with 4% paraformaldehyde (PFA). The removed brain was immersed in 4% PFA overnight, and subsequently in 30% sucrose for 48 h at 4°C. After the brain had been cut into coronal 30-μm sections, H_2_O_2_-inactivation of endogenous peroxidase activity was performed for immunohistochemistry, followed by treatment with 2% bovine serum albumin (BSA) in PBS-0.2% Triton X-100 for 60 min at room temperature to block non-specific protein binding. The sections were incubated with a rabbit monoclonal antibody against c-Fos (Cell Signaling Technology, Beverly, MA, United States) and a mouse monoclonal antibody against FOX3 overnight at 4°C. After incubation with a HRP-conjugated secondary antibody, the sections were then treated with 3,3′-diaminobenzidine (DAB) for immunohistochemistry. An Alexa Fluor 594-labeled donkey polyclonal antibody against rabbit IgG (H + L), F (ab’)2 fragment and an Alexa Fluor 488-labeled donkey polyclonal antibody against goat IgG F (ab’)2 fragment were used for immunofluorescence. Fluorescence was observed using a Nikon C2 Si confocal microscope system.

### Western Blotting

Frozen brain sections (30 μm) were stained with toluidine blue, and then the PVN was collected by laser microdissection (LMD) in accordance with the mouse brain map. The collected PVNs were lysed in 2 x SDS sample buffer (EzApply, ATTO, Tokyo, Japan) and boiled at 100°C for 5 min. The boiled lysate was then subjected to sodium dodecyl sulfonyl sulfate polyacrylamide gel electrophoresis (SDS-PAGE) using a 5–20% e-PAGEL gradient gel (ATTO, Tokyo, Japan), and blotted onto polyvinylidene fluoride (PDNF) membranes. The membranes were blocked with 2% skim milk in PBS−0.1% Tween 20 for 60 min at room temperature, and then incubated with a rabbit monoclonal antibody against LRRK2 (MJFF2, Abcam, Cambridge, MA, United States), a rabbit monoclonal antibody against p44/42 MAPK (Erk1/2, Cell Signaling Technology, Beverly, MA, United States), a rabbit monoclonal antibody against phospho-p44/42 MAPK (Thr202/Tyr204, Cell Signaling Technology, Beverly, MA, United States), and a rabbit monoclonal antibody against glyceraldehyde-3-phosphate dehydrogenase (GAPDH, Cell Signaling Technology, Beverly, MA, United States) overnight at 4°C. After incubation with the secondary antibody (HRP-conjugated donkey anti-rabbit IgG antibody) for 30 min at room temperature, protein bands were detected using a Luminata Crescendo (Merck Millipore, Guyancourt, France). The signals were captured by an Odyssey Fc Imaging System (LI-COR, Lincoln, NE, United States) followed by quantification of the signals with Image Studio 5.0.

### Quantitative Reverse Transcription (RT)-PCR

The PVN collected by LMD was homogenized in TRIzol Reagent (Thermo Fisher Scientific, Waltham, MA, United States). RNA was isolated in accordance with the manufacturer’s instructions. cDNA synthesis was performed using a ThermoScript RT-PCR System (Thermo Fisher Scientific, Waltham, MA, United States). Real-time PCR was performed using SYBR Green PCR Master Mix and a 7500 Real-Time PCR System (Thermo Fisher Scientific, Waltham, MA, United States). PCR primers used were as follows: CRF forward 5′−TGATCCGCATGGGTGAAGAAT ACTTCCTC−3′ and reverse 5′−CCCGATAATCTCCATCAGT TTCCTGTTGCTG−3′, and GAPDH forward 5′−GAGGCCG GTGCTGAGTATGTCGTG−3′ and reverse 5′−TCGGCAGAA GGGGCGGAGAT−3′. The threshold cycle (Ct) value was normalized by reference to GAPDH.

### Measurement of Adrenocorticotropic Hormone

Blood samples were collected into tubes with EDTA 1 h after the stress exposure, and plasma samples were obtained by centrifugation at 1,500 rpm for 10 min at 4°C. The amounts of plasma ACTH were measured using an ACTH Rat/Mouse ELISA Kit (Phoenix Pharmaceuticals, Burlingame, CA, United States) in accordance with the manufacturer’s protocol.

### Measurement of Metabolism

Body weight and metabolism were measured daily using a metabolic cage for 3 days in a row following habituation to the cage for 7 days. The amounts of food intake, feces, water intake, and urine volume were measured as weight in grams.

### Intestinal Transit Assay

Intestinal motility in mice was evaluated from the distribution of orally administered fluorescein isothiocyanate (FITC)-conjugated dextran. Mice were exposed to the restraint stress just before oral administration of the dextran. After transportation of the dextran along the gastrointestinal tract for 3 h, the whole gut was collected after euthanasia. The small intestine and the colon were equally cut into ten segments and four segments, respectively. The fluorescence intensity of each piece was measured using FLUOstar OPTIMA (BMG LABTECH, Saitama, Japan). The intestinal geometric center was used for evaluation of the distribution of dextran, which was calculated using the following equation:

geometric center=⁢∑(counts per segment×segment number/total count)

### Bead Expulsion Assay

Colonic motility was evaluated by the bead expulsion assay. Following anesthesia with isoflurane (Wako, Osaka, Japan), a zirconia bead (3-mm diameter) was inserted into the rectum at a distance of 2-cm from the anal verge. The time required for expulsion of the bead beyond the anal verge was then measured. Each mouse was subjected to the same experiment again after the restraint stress. For comparison between WT and KO mice, we calculated the reduction in time until excretion of the bead after the restraint stress.

### Whole-Mount Staining of the Longitudinal Muscle-Myenteric Plexus

The mouse colon was opened and pinned on a silicone-sheeted glass dish. Each preparation was fixed overnight with 4% PFA, and then the mucosa, submucosa, and circular muscle layer were removed under a dissecting microscope. Preparations were incubated in PBS-1% Triton X-100 for 30 min, followed by incubation with rabbit monoclonal antibody against c-Fos (Cell Signaling Technology, Beverly, MA, United States), goat polyclonal antibody against choline acetyltransferase (ChAT, Merck Millipore, Guyancourt, France), and goat polyclonal antibody against neuronal nitric oxide synthase (nNOS, Abcam, Cambridge, MA, United States) for 24 h at 4 C. And subsequently with Alexa Fluor 594-labeled donkey polyclonal antibody against rabbit IgG (H + L), F (ab’)_2_ fragment (Cell Signaling Technology, Beverly, MA, United States) or Alexa Fluor 488-labeled donkey polyclonal antibody against goat IgG F (ab’)_2_ fragment (Abcam, Cambridge, MA, United States) for 3 h at 4°C. Nuclear staining was performed with 2′-(4-ethoxyphenyl)-5-(4-methyl-1-piperazinyl)-2,5′-bi-*1H*-benzimidazole (Hoechst 33342, Kumamoto, Japan). Fluorescence was observed using a Nikon C2 Si confocal microscope system.

### Statistical Analysis

All data are expressed as means + SEM. Significance of differences was assessed by Student’s *t* test for two comparisons and two-way ANOVA for multiple comparisons. Differences at *p* < 0.05 were considered to be significant. Sample size *n* represents the number of animals.

## Results

### Attenuated Reactivity of the PVN in Response to Restraint Stress in LRRK2-KO Mice

**FIGURE 1:**
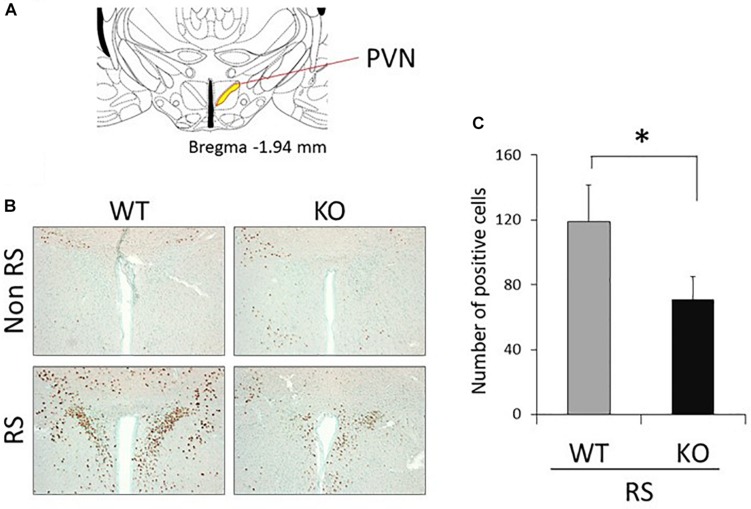
Immunohistochemistry using an antibody against c-Fos in the PVN. **(A)** Section representing a level –1.94 mm posterior to the bregma. Yellow area indicates the PVN of the hypothalamus. **(B)** Representative immunohistochemistry using an antibody against c-Fos in the PVN of WT and LRRK2-KO mice in the absence or presence of restraint stress (RS). Methyl green was used for counter staining. Scale bar = 100 μm. **(C)** The total number of c-Fos-positive cells counted in the PVN. *n* = 5 per group. In all graphical representations, data are expressed as mean + SEM and were assessed by Student’s*t* test (WT vs. KO), ^∗^*p* < 0.05.

**FIGURE 2:**
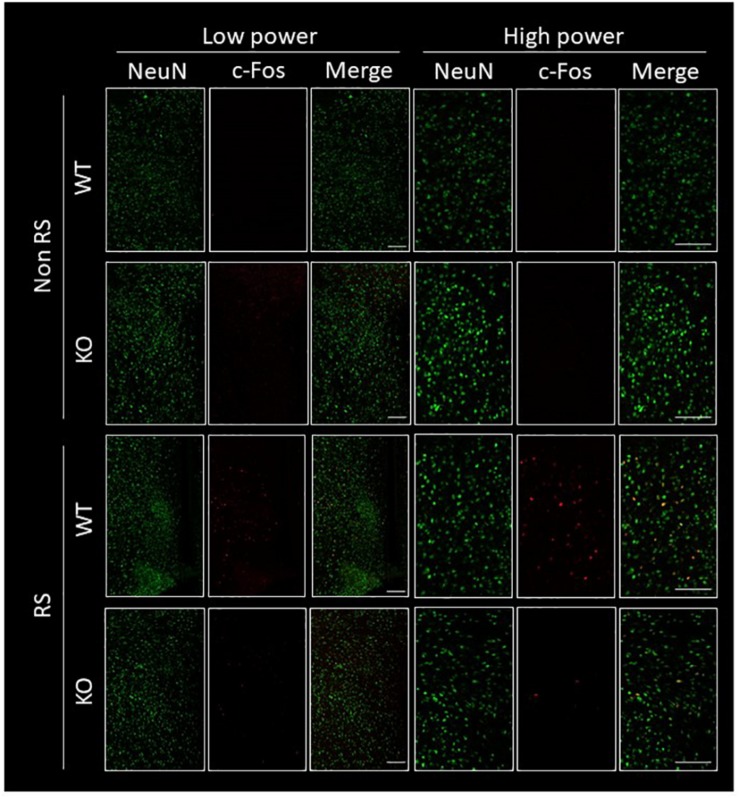
Immunofluorescence of c-Fos positive cells in the PVN. Confocal images of double immunostaining using antibodies against NeuN and c-Fos in the PVN of WT and LRRK2-KO mice in the absence or presence of restraint stress (RS). Scale bar = 100 μm.

The PVN of the hypothalamus is one of the major regions involved in reactivity to stress, and restraint is known to be a major stressor that activates neurons (Kwon et al., 2006; Zheng et al., 2009; Rutherford et al., 2011; Campos-Rodríguez et al., 2013). Therefore, to explore the association between LRRK2 and stress, expression of c-Fos (a marker of neuronal activation) under restraint stress was analyzed in LRRK2-KO mice. Activated PVN neurons were clearly visualized by immunostaining with an antibody against c-Fos only after restraint stress in both WT and KO mice (Figures 1A,B). Interestingly, the number of c-Fos positive neurons was strikingly decreased in KO mice relative to WT mice, indicating attenuation of neuronal reactivity to the stress in KO mice (Figure 1C). We confirmed that reactivity for c-Fos after the stress was detected only in neurons under our restraint stress conditions (Figure 2).

### Disruption of the ERK Signaling Pathway in the PVN of LRRK2-KO Mice

**FIGURE 3:**
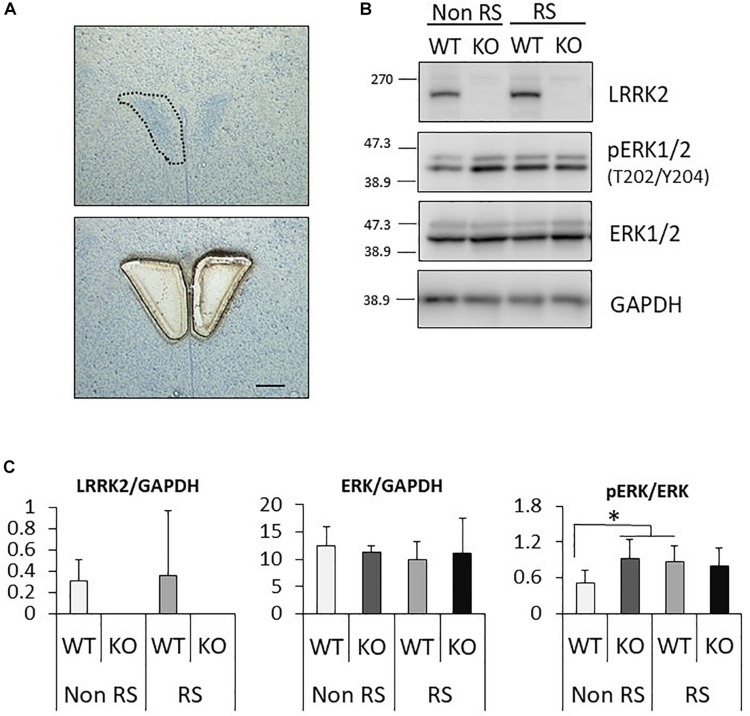
Analysis of the PVN signaling pathway by Western blotting. **(A)** Representative toluidine blue staining at the level of the posterior PVN before (upper panel) and after (lower panel) LMD. Scale bar = 100 μm. **(B)** Representative images of Western blotting using antibodies against LRRK2, pERK1/2 (Thr202/Tyr204), ERK1/2, and GAPDH in LRRK2-KO mice. **(C)** Quantitative analysis of the density of protein bands. Each signal intensity was normalized against that of GAPDH or pan-ERK1/2. *n* = 5 per group. In all graphical representations, data are expressed as mean + SEM and were assessed by two-way ANOVA, ^∗^*p* < 0.05.

**FIGURE 4:**
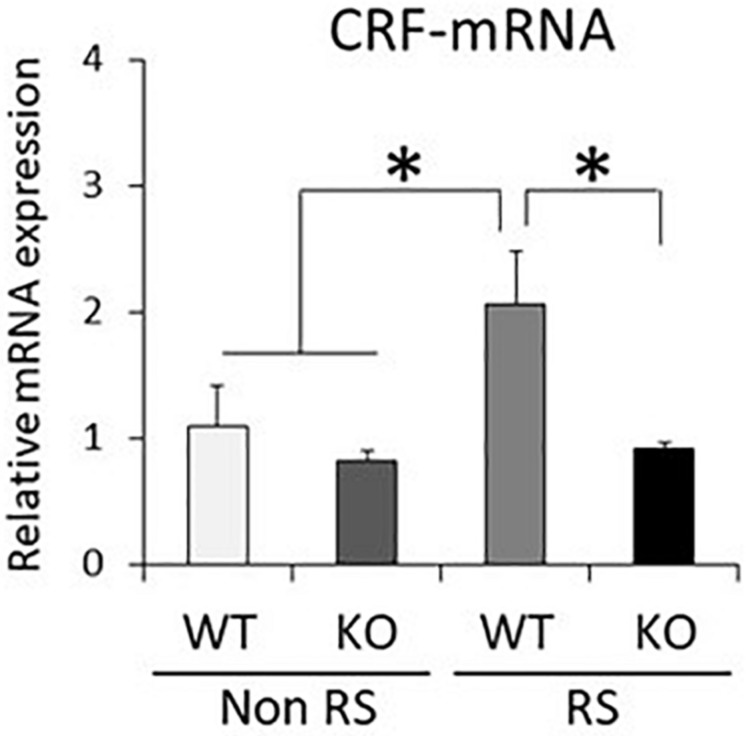
Down-regulated CRF-mRNA level in the PVN in the presence of restraint stress in LRRK2-KO mice. Total mRNA prepared from microdissected PVN in the absence or presence of restraint stress was subjected to quantitative PCR using a specific primer for CRF. *n* = 6 per group. In all graphical representations, data are expressed as mean + SEM and were assessed by two-way ANOVA, ^∗^*p* < 0.05.

It has been suggested that ERK signaling is the pathway affected by LRRK2 through its kinase activity, and that it is also crucial for stress signaling under stress (Monje et al., 2005; Liou et al., 2008). To investigate whether the ratio of phosphorylated ERK1/2 is changed in LRRK2-KO mice, the protein levels of LRRK2, pERK1/2, ERK1/2, and GAPDH in the PVN were quantified by Western blotting. A PVN-protein lysate was extracted from pieces of tissue collected by LMD (Figure 3A). Western blotting analysis detected specific bands at each of the predicted molecular weights in both WT and KO mice, and equivalent protein loading was confirmed by comparison with the signal intensity of GAPDH (Figure 3B). Unchanged levels of LRRK2 and ERK1/2 total protein after the stress were confirmed by GAPDH normalization (Figure 3C). It is noteworthy that the ratio of ERK1/2 phosphorylation was significantly increased in LRRK2-KO mice even in the absence of stress. Moreover, phosphorylation of ERK1/2 was increased by the stress in WT mice, but not in KO mice. These results indicated that LRRK2 deficiency dramatically affects the phosphorylation of ERK1/2 in the PVN, suggesting that LRRK2 acts as a molecule in stress pathways. Corticotropin releasing hormone (CRF) is the most important transmitter involved in the regulation of stress responses within the PVN (Lightman, 2008). Therefore, we investigated whether CRF expression was increased following restraint stress. Samples dissected from the PVN were subjected to quantitative RT-PCR using a CRF primer and a GAPDH primer as a target and internal control, respectively. Restraint stress induced up-regulation of the CRF-mRNA level in WT mice following the stress (Figure 4). On the other hand, the mRNA level in KO mice was not up-regulated following the stress.

### Down-Regulation of ACTH Reactivity in LRRK2-KO Mice

**FIGURE 5:**
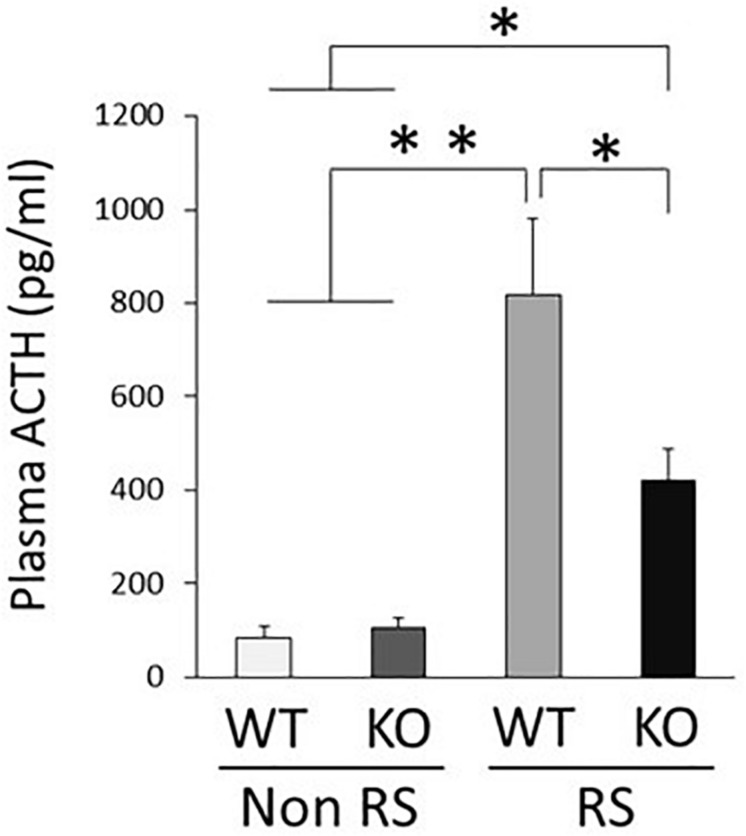
Lower plasma level of ACTH in the presence of restraint stress in LRRK2-KO mice. Quantitative analysis of plasma ACTH by ELISA in the absence or presence of restraint stress. *n* = 6 per group. In all graphical representations, data are expressed as mean + SEM and were assessed by two-way ANOVA, ^∗∗^*p* < 0.01, ^∗^*p* < 0.05.

An increase in the plasma level of ACTH is a major response following PVN activation caused by stress. Quantification of plasma ACTH revealed small amounts of ACTH in the absence of stress, without any significant difference between WT and KO mice (Figure 5). This increase in the level of ACTH was detected in the presence of stress in both WT and KO mice, but was significantly lower in the latter than in the former, in line with decreased activation of the PVN.

### Aberrant Gastrointestinal Response to Restraint Stress in LRRK2-KO Mice

**FIGURE 6:**
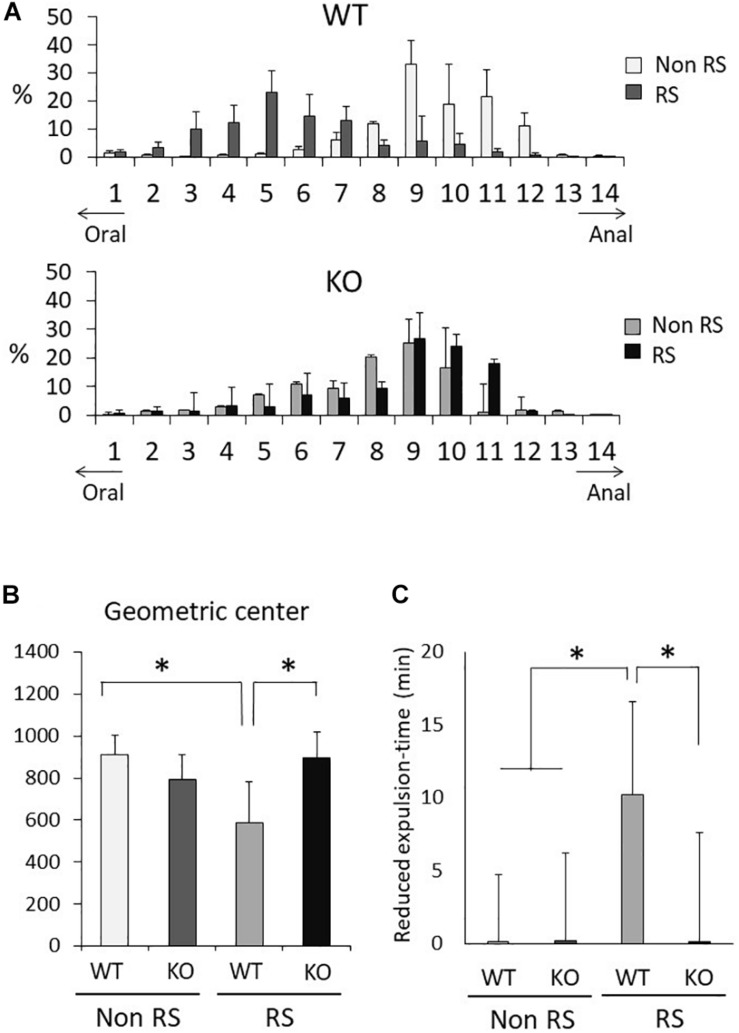
Aberrant gastrointestinal response to restraint stress in LRRK2-KO mice. **(A)** Intestinal transit assay using FITC-conjected dextran in the absence or presence of restraint stress. Histogram represents the proximal to distal distribution of orally administered FITC-conjugated dextran along the gastrointestinal tract (1–14). *n* = 6 per group. The data represent the percentage distribution relative to the whole amount of dextran. **(B)** Intestinal geometric center of the distribution of dextran. **(C)** Bead expulsion assay for analysis of colonic motility. The time required to expel the bead was measured. Data represent the reduction in the time period until excretion due to stress. *n* = 6 per group. In all graphical representations, data are expressed as mean + SEM and were assessed by two-way ANOVA for multiple comparison and Student’s *t* test for two comparison (WT vs. KO), ^∗^*p* < 0.05.

It is known that gastrointestinal motility is affected by stress, sometimes resulting in functional gastrointestinal disorder (FGID). To investigate gastrointestinal motility under restraint stress, we performed the intestinal transit assay using FITC-conjugated dextran as an indicator of upper gastrointestinal motility and the bead expulsion assay as an indicator of colonic motility. Before these assays, the amounts of food intake, feces production, water intake, and urine volume per day were measured using metabolic cages. Accompanied by normal weight fluctuation, the metabolism of LRRK2-KO mice was comparable to that of WT mice (Supplementary Figure S1). The intestinal transit assay demonstrated a gradient distribution throughout the gastrointestinal tract with a peak point (Figure 6A). The peak of dextran distribution shifted to the oral side in the presence of stress relative to that in its absence, indicating delayed upper gastrointestinal motility in WT mice. The geometric center of dextran distribution also showed that upper gastrointestinal motility was delayed under restraint stress in WT mice (Figure 6B). In contrast, no alteration in the distribution of dextran was observed in KO mice, as indicated by an unchanged geometric center in the presence of stress. The bead expulsion assay for analysis of colonic motility revealed that the time required to excrete a bead that had been inserted into the rectum was shorter in the presence of stress than its absence in WT mice, indicating that the stress had induced colonic dysmotility (Figure 6C). This stress-induced colonic dysmotility was not observed in KO mice, suggesting disruption of stress reactivity in terms of colonic as well as upper gastrointestinal motility in KO mice.

### Attenuation of c-Fos Immunoreactivity of Enteric Excitatory Neurons in Response to Restraint Stress in LRRK2-KO Mice

**FIGURE 7:**
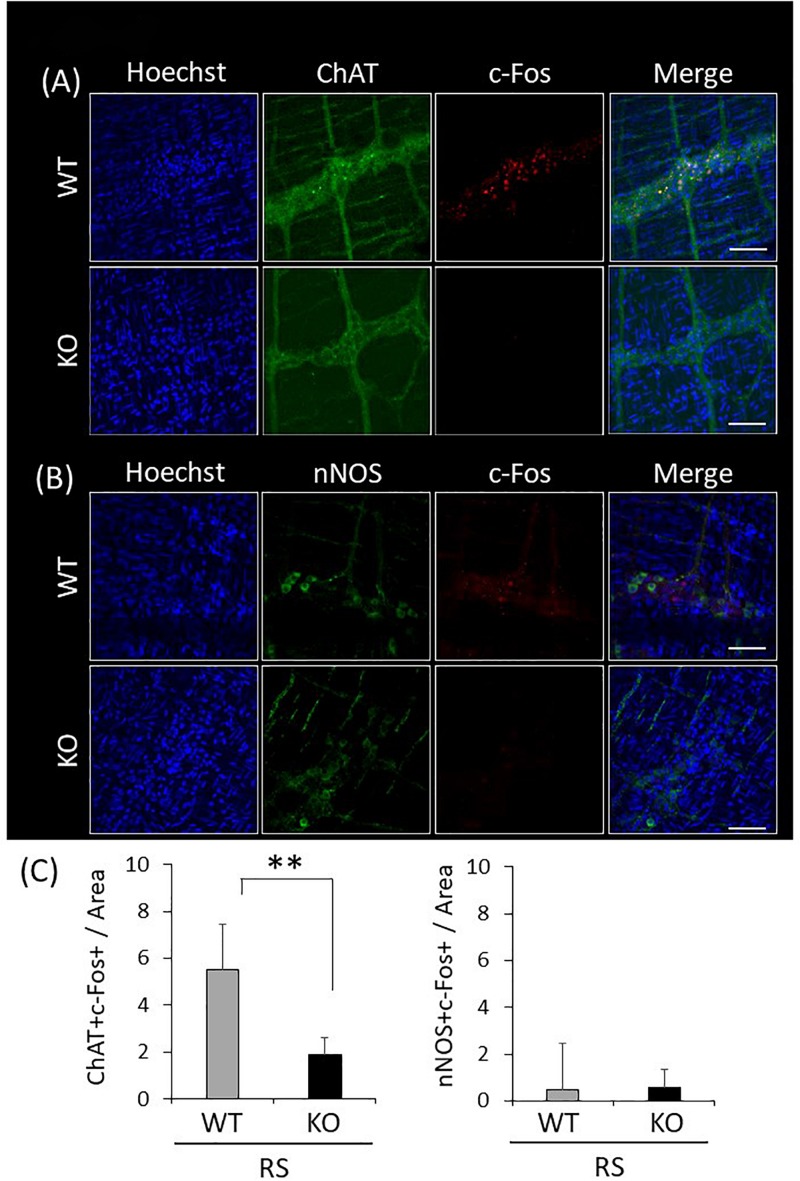
Attenuated reactivity of enteric excitatory neurons in response to restraint stress in LRRK2-KO mice. **(A)** Confocal images of double immunostaining using antibodies against ChAT and c-Fos in the LMMP of the colon after restraint stress. Nuclei were visualized using Hoechst 33342. Scale bar = 100 μm. **(B)** Confocal images of double immunostaining using antibodies against nNOS and c-Fos in the LMMP of the colon after restraint stress. **(C)** Graphical data represent the number of double-immunopositive cells (ChAT^+^/c-Fos^+^ or nNOS^+^/c-Fos^+^) per area in the colon. *n* = 6 per group in the presence of restraint stress. In all graphical representations, data are expressed as mean + SEM and were assessed by Student’s *t* test (WT vs. KO), ^∗∗^*p* < 0.01.

Since gastrointestinal motility is orchestrated by enteric neuronal circuits concurrently with signals from the CNS, enteric neurons activated in response to restraint stress were characterized by immunostaining for LMMP using antibodies against ChAT or nNOS, which visualize enteric excitatory or inhibitory neurons, respectively, and c-Fos. Activated neurons were simultaneously visualized using the antibody against c-Fos. This revealed that excitatory neurons were predominant in enteric neuronal ganglia, whereas inhibitory neurons were sparse (Figures 7A,B). Immunoreactivity for c-Fos was evident in the nuclei of neuronal cells only after the stress, and c-Fos positivity was more abundant in ChAT-positive than in nNOS-positive cells (Figure 7C). In KO mice, double-immunopositive cells (ChAT^+^/c-Fos^+^) were significantly decreased in comparison with WT mice; however, there was no difference in the number of nNOS^+^/c-Fos^+^ cells between WT and KO mice. These results suggested that disruption of gastrointestinal dysmotility in LRRK2-KO mice is due to lower activation of enteric excitatory neurons.

## Discussion

In this study, we investigated the role of LRRK2 in the PVN stress pathway and the resulting gastrointestinal dysmotility for the first time. LRRK2-KO mice exhibited alleviation of gastrointestinal dysmotility caused by restraint stress, along with disruption of the ERK signaling pathway. Furthermore, peripheral stress responses – in terms of an increased plasma ACTH level and gastrointestinal dysmotility – were suppressed in KO mice. Our study has highlighted LRRK2 as a new candidate molecule regulating PVN reactivity in stress and gastrointestinal dysmotility.

It has been shown that the PVN plays a crucial role in the brain as a stress sensor (Herman, 2012). Given that LRRK2-KO mice exhibited a lower number of c-Fos-positive neurons in the presence of restraint stress than was the case in WT mice, there is a possibility that LRRK2 up-regulates neuronal reactivity in the PVN in response to stress (Figure 1C). Transgenic mice harboring LRRK2 mutation (R1441G, R1441C, G2019S, I2020T) might be helpful for understanding the association between stress and PD. We further revealed that abundant CRF expression following the stress was not observed in KO mice (Figure 3). Since CRF is the important transmitter involved in stress responses within the PVN, we considered that the loss of LRRK2 signaling ensures disturbance of stress responses (Wamsteeker et al., 2013; Jiang et al., 2018). Interestingly, linkage disequilibrium analysis has revealed that CRF receptor 1 is a molecule associated with PD (Oliveira et al., 2004). Accordingly, there is a possibility that the CRF-related pathway is associated with not only stress signaling but also PD development in tandem with LRRK2. From another viewpoint, the lower neuronal reactivity in the PVN of LRRK2-KO mice might be due to defective release and/or binding of CRF, as LRRK2 has a role in vesicle trafficking and receptor trafficking (Dodson et al., 2012; Matta et al., 2012; MacLeod et al., 2013; Beilina et al., 2014; Cho et al., 2014; Yun et al., 2015; Steger et al., 2016; Rassu et al., 2017). In addition to neurons expressing CRF, the PVN contains some types of neuron expressing thyrotropin-releasing hormone, arginine vasopressin, oxytocin, or somatostatin (Biag et al., 2012). It is problematic to clarify the expression of LRRK2 in the PVN because the reactivity of anti-LRRK2 antibody is limited, particularly under the conditions used for immunostaining (Davies et al., 2013). Only mRNA data are available with regard to LRRK2 expression in the PVN in rat and mouse brain (Simón-Sánchez et al., 2006; Taymans et al., 2006). Genetic approaches, such as the use of LRRK2-reporter mice, could be one way to resolve this issue.

In terms of c-Fos immunoreactivity, astroglia also have positive immunoreactivity under stress conditions (Fuente-Martín et al., 2016). Although the c-Fos-positive cells were neurons in our stress model, astroglia are another type of regulatory cell that can control stress reactivity through PVN neurons.

A number of intercellular signaling events have been attributed to LRRK2 kinase activity, and in addition LRRK2 acts as a scaffolding protein by interacting with ASK1, MKK3/6, and p38MAPK (Lobbestael et al., 2012; Yoon et al., 2017). Since the level of phosphorylation at Ser1292 has been proposed as a direct marker of LRRK2 kinase activity, we tried to detect phosphorylated LRRK2 (Ser1292) using PVN samples but were unable to find a clear specific band. Our present results were unable to indicate whether kinase activity or scaffolding function is substantially affected. Further *in vitro* experimental data will be necessary to clarify the predominant role of LRRK2 in the stress-signaling pathway.

LRRK2-KO mice also showed lower reactivity to stress and higher expression of phosphorylated ERK1/2 in the PVN, which appeared to suppress any stress-induced effects. As protein kinase A (PKA) activation is required for phosphorylation of ERK1/2 and LRRK2 negatively regulates PKA, augmented phosphorylation of ERK1/2 could be due to higher PKA activity caused by LRRK2 depletion (Impey et al., 1998; Zanassi et al., 2001; Chen et al., 2010; Parisiadou et al., 2014; Greggio et al., 2017; Russo et al., 2018). Kubo et al. (2016) have reported a higher degree of ERK1/2 phosphorylation in B2 B-cells of LRRK2-KO mice, thus highlighting the importance of further investigation into the interaction between LRRK2 and ERK1/2 (Kubo et al., 2016).

The neuronal activity of the PVN is regulated via the negative feedback action of corticosterone. Osterlund et al. have reported that ERK1/2 activation was inhibited by the tonic but not phasic feedback action of corticosterone (Osterlund et al., 2011). They suggested that ERK1/2 activation of the PVN may be associated with altered activity of stress-dependent neuronal inputs to the PVN, and that alteration of stress-dependent intracellular signaling mechanisms within those neurons might be associated with ERK1/2 activation. Therefore, this feedback mechanism might induce persistent activation of ERK1/2 and defective ERK1/2 activation in KO mice.

Although restraint stress suppresses upper gastrointestinal motility and promotes colonic motility, the response of LRRK2-KO mice to this form of stress was deficient and atypical (Taché et al., 1999). Although the lower ACTH level in KO mice indicates disruption of the hypothalamic-pituitary-adrenal (HPA) axis, other pathways can transmit stress signals from the brain to peripheral organs. The autonomic nervous pathway, prolactin release, gonadal steroid release, and IL-6 release may also have possible roles in regulation of the stress reaction (Herman et al., 2016). As the HPA axis plays an important role in stress responses, it may be necessary to consider several aspects of the stress reactive pathway, in order to integrally understand its association with LRRK2.

Activation of cholinergic enteric neurons in the presence of stress was suppressed in KO mice relative to WT mice, suggesting that disruption of the stress response in KO mice, in terms of altered gastrointestinal motility, was due to reduced reactivity of cholinergic neurons. Recently, a PET study has shown that the abnormal cholinergic function of individuals with LRRK2 mutations, both with or without PD, was due to increased rates of acetylcholinesterase hydrolysis (Liu et al., 2018). In addition, Higashi et al. (2007) have revealed that LRRK2 is expressed in ChAT-positive, but not nNOS-positive neurons (Higashi et al., 2007). These reports indicate that LRRK2 shows higher expression in cholinergic than in nitrergic neurons, and that the cholinergic system might be more affected by LRRK2 aberration. Our recent study showed that, like brain neurons, enteric neurons also express LRRK2 (Maekawa et al., 2016a, b). However, the characteristics of these neurons have not been revealed. Identification of the cells predominantly expressing LRRK2 in the enteric nervous system (ENS) could be the key to elucidating the mechanism of dysmotility in LRRK2-KO mice. More specific characterization of LRRK2-expressing cells in the ENS would help to shed light on cholinergic dysfunction in PD.

Enteric glial cells (EGCs) have emerged as another component of gastrointestinal motility control (Neunlist et al., 2014). Fujikawa et al. (2015) have investigated the association between EGCs and gastrointestinal dysmotility in maternally separated rats (Fujikawa et al., 2015). Also, a lower level of glial fibrillary acidic protein (GFAP) phosphorylation has been observed in colonic biopsy samples from PD patients (Clairembault et al., 2014). Given that neurons and EGCs comprise a huge complex circuit, and that astroglia express LRRK2 in the CNS, it is important to further analyze the ENS structure of LRRK2-KO mice, including their degree of EGC complexity (Coelho-Aguiar et al., 2015).

Gastrointestinal dysfunction is common in both idiopathic and familial PD (Marras et al., 2011). However, Gaiq et al. (2014) have recently reported that LRRK2 G2019S patients show no symptomatic gastrointestinal dysmotility (Gaiq et al., 2014). Since our study using LRRK2-KO mice also demonstrated normal gastrointestinal motility in the absence of stress, some additional risk factor, for example gene mutation, or environmental and/or psychological stress, might be necessary for induction of gastrointestinal dysfunction in LRRK2-related PD.

Taken together, our present findings suggest that LRRK2 is a newly recognized molecule regulating the stress pathway in the PVN, thus playing a role in stress-related gastrointestinal dysmotility. These findings will be of help for further studies of LRRK2 in the context of both the gut and the brain, especially in relation to PD.

## Data Availability

All datasets generated for this study are included in the manuscript and/or the Supplementary Files.

## Ethics Statement

All procedures had been approved by the Animal Experimentation and Ethics Committee of Kitasato University.

## Author Contributions

TM, HT, and MI performed the experiments with support from FK, RK, MK, and TI. TM designed the study and wrote the manuscript with support from TI. All authors have read and approved the manuscript.

## Conflict of Interest Statement

The authors declare that the research was conducted in the absence of any commercial or financial relationships that could be construed as a potential conflict of interest.
