# Supplementary material for: Leucine-Rich Repeat Kinase 2 Is Associated With Activation of the Paraventricular Nucleus of the Hypothalamus and Stress-Related Gastrointestinal Dysmotility
Source: Front Neurosci. 2019 Aug 29;13:905. doi: 10.3389/fnins.2019.00905 (PMC6727664; doi:10.3389/fnins.2019.00905)
Supplement: Supplementary file 1 [file Data_Sheet_1.PDF]

Supplementary Material

Leucine-Rich Repeat Kinase 2 is Associated with Activation of the Paraventricular Nucleus of the Hypothalamus and Stress-Related Gastrointestinal Dysmotility

Tatsunori Maekawa[1*], Hiromichi Tsushima[1,2], Fumitaka Kawakami[1], Rei Kawashima[1], Masaru Kodo[1], Takafumi Ichikawa[1]
[1]Department of Regulation Biochemistry, Graduate School of Medical Sciences, Kitasato University, Sagamihara, Kanagawa Japan.
[2]Department of Behavioral Medicine, Tohoku University Graduate School of Medicine, Sendai, Miyagi, Japan.

[*] **Correspondence:**
Dr. Tatsunori Maekawa
maekawa@kitasato-u.ac.jp

Supplementary Figure Legend

Supplementary Figure 1 | No abnormalities of metabolism in LRRK2-KO mice. Weight fluctuation and metabolism in terms of food intake, feces production, water intake, and urine volume excreted daily were measured using metabolic cages. $n = 6$ per group.
