# Supplementary figures and images for: Leucine-Rich Repeat Kinase 2 Is Associated With Activation of the Paraventricular Nucleus of the Hypothalamus and Stress-Related Gastrointestinal Dysmotility
Source: Front Neurosci. 2019 Aug 29;13:905. doi: 10.3389/fnins.2019.00905 (PMC6727664; doi:10.3389/fnins.2019.00905)

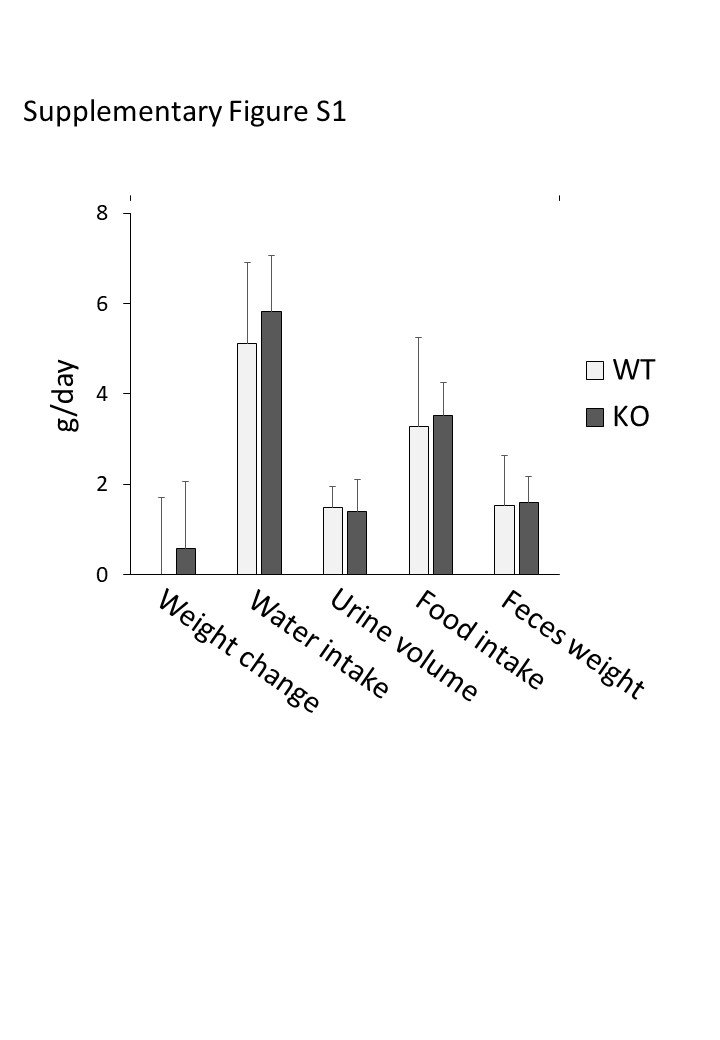

Supplement: Supplementary file 2 [file Image_1.JPEG]
